# A study on the falls factors among the older adult with cognitive impairment based on large-sample data

**DOI:** 10.3389/fpubh.2024.1376993

**Published:** 2024-06-14

**Authors:** Changying Wang, Yunwei Zhang, Jin Wang, Lingshan Wan, Bo Li, Hansheng Ding

**Affiliations:** ^1^Shanghai Health Development Research Center, (Shanghai Medical Information Center), Shanghai, China; ^2^Minhang Hospital, Fudan University, Shanghai, China

**Keywords:** older adult, correlative factors, falls, MCI, dementia

## Abstract

**Introduction:**

This study explored the correlative factors of falls among the older adult with cognitive impairment, to provide distinct evidence for preventing falls in the older adult with cognitive impairment compared with the general older adult population.

**Methods:**

This study was based on a cross-sectional survey, with an older adult population of 124,124 was included. The data was sourced from the Elderly Care Unified Needs Assessment for Long-Term Care Insurance in Shanghai. Binary and multivariable logistic regression analyses were conducted sequentially on the correlative factors of falls. Multivariable logistic regression was performed on variables that were significant, stratified by cognitive function levels.

**Results:**

The incidence of fall in the past 90 days was 17.67% in this study. Specific variables such as gender (male), advanced age (≥80), residence with a elevator (or lift), mild or moderate disability, quality of sleep (acceptable/poor) were negatively correlated with falls, while higher education level, living alone, residence with indoor steps, unclean and untidy living environment, MCI or dementia, chronic diseases, restricted joints, impaired vision, and the use of diaper were positively correlative factors of falls. Comparing with older adult with normal cognitive functions, older adult with dementia faced a higher risk of falling due to accessibility barrier in the residence. For general older adults, less frequency of going outside and poor social interactions were positively correlated with falls, while for older adult with cognitive impairments, going outside moderately (sometimes) was found positively correlated with falls. Older adults with cognitive impairments have increased fall risks associated with chronic diseases, restricted joints, and the use of diaper. The risk of falling escalated with the greater number of chronic diseases.

**Discussion:**

For older adult with cognitive impairments, it is advisable to live with others. Additionally, creating an accessible living environment and maintaining the cleanness and tidiness can effectively reduce the risk of falls, particularly for those with MCI or dementia. Optimal outdoor activity plans should be developed separately based on the cognitive function of older adults. Older adult with dementia who have comorbidities should be paid special attention in fall prevention compared to the general older adult population.

## 1 Introduction

The World Health Organization (WHO) defines falls as: an event which results in a person coming to rest inadvertently on the ground or floor or other lower level (1). Falls are a major public health concern, and as a leading cause of unintentional death, falls are second only to road traffic injuries worldwide. According to estimates by WHO, 684,000 fatalities annually can be attributed to falls, with more than 80% of these deaths occurring in low- and middle-income countries. In developed countries, falls are also a very common cause of accidental death (2–5).

Among people who fall, the subgroup of adults aged 60 and above account for the greatest number of fatal falls (1). Falls not only have a crippling impact on the quality of life of the older adult both physically and psychologically, but also impose a serious economic and care burden on families and society (6, 7). In China, falls are the leading cause of traumatic fractures, injury-related medical visits and death among the older adult (8). Older adults who have experienced a fall face significant risks such as a decline in quality of life, the need for long-term care, and the possibility of staying in a nursing facility (1). Falls can also have a negative impact on individual’s physical activity (9).

Dementia refers to a range of related diseases that affect memory, thinking, and the activity of daily living (ADL) (10). It is characterized by the gradual loss of cognitive functions such as memory and orientation, as well as psychological and behavioral symptoms, and it is also accompanied by a weakening of physical functions (11). Mild cognitive impairment (MCI) is considered a transitional phase between the normal cognitive declines associated with aging and dementia, with progressive deterioration in memory and other cognitive functions, but not yet meeting the diagnostic criteria for dementia (12, 13). As the symptoms of cognitive impairment progress, it can lead to people becoming more unsteady and prone to fall (14). Older adult with cognitive impairment (15)-include MCI and dementia, have a significant increased risk of falling compared with the general older adult group (16–18), and are at a higher risk of suffering from serious injuries (19). It is reported that most falls were not caused by a single risk factor, but by the interaction of several factors which determine the outcome (20). The approach to fall prevention targeting the risk factors may vary between early-stage cognitive impairment and those with more advanced conditions, given the progression of cognitive impairment and functional decline (21). Therefore, identifying and addressing a series of distinct factors related to falls among older adults with MCI and dementia, as compared to general older adult population, is of particular importance.

At present, over 55 million individuals have dementia worldwide, with approximately 10 million new cases emerging annually. Moreover, over 60% of these individuals reside in low- and middle-income countries (10). In China, the prevalence of cognitive impairment is 28.1% according to the Chinese Longitudinal Healthy Longevity Survey (CLHLS) (22). As the expected growth of older adult with cognitive impairment, it has implications for falls and the related consequences. Thus, based on large-sample cross-sectional data, this study explored the possible correlative factors of falls among the older adult with MCI and dementia, and focused on factors related to falls in older adults with CI compared to older adults without CI, and intended to provide more targeted evidences.

## 2 Methods

### 2.1 Study design

This study was based on a cross-sectional survey, with a population of older adults aged 60 and above, who voluntarily applied for and received the Elderly Care Unified Needs Assessment for Long-Term Care Insurance in Shanghai, China from January to May 2023. The research protocol, bearing the reference number 2020001, obtained official approval from the Ethics Committee of the Shanghai Health Development Research Center. Before the assessment began, all applicants signed an informed consent form. For those older adult individuals who were too frail and unable to sign can have their guardian sign to confirm on their behalf. Data analysis was carried out on information related to this study and did not involve personal information including names, ID numbers, home addresses, etc.

### 2.2 Inclusion criteria

The exclusive criteria for participants were: (1) severe disability; (2) diagnosed as dementia by a professional psychiatrist. Previous studies indicated that the use of psychotropic medications had association with falls (23–25), with side effects such as dizziness, orthostatic hypotension, and decreased alertness (26), thus older adult with a definitive diagnosis of dementia were excluded from this study; (3) with life-threatening diseases, which was confirmed by the family doctor based on older adult’s medical records. For example, end-stage of cancer.

**Figure 1:**
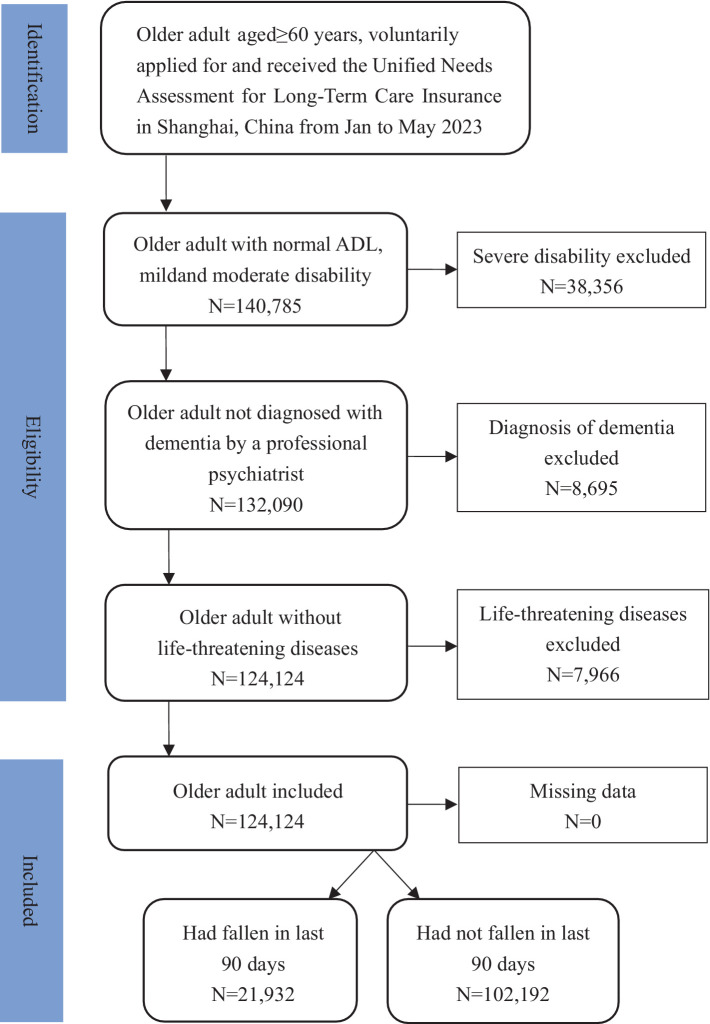
Flow chart of participants selection.

Finally, 124,124 individuals were included. Fall was measured by family doctors according to medical records within the last 90 days, which referenced International Resident Assessment Instrument (InterRAI) standards (27). Of these, 21,932 individuals, accounting for 17.67%, had experienced a fall within the last 90 days, while 102,192 individuals, representing 82.33%, had not experienced a fall within the last 90 days (Figure 1).

### 2.3 Data collection

The data in this study was gathered from the Elderly Care Unified Needs Assessment for Long-Term Care Insurance in Shanghai. Assessors must complete and pass a standardized city-wide uniform training program before they are qualified to conduct the home visit. The assessment team for home visit should be composed of at least two professionals: one with experience in older adult care, nursing, or social work, and the other is family doctor who should be familiar with the older adult’s health conditions. During the assessment, the presence of a family member, caregiver, or guardian of the older adult is required, to help provide information and assist with the assessment work. “Elderly Care Unified Needs Assessment Form” was employed in home visit assessment, data was collected by the assessment team for home visit and inputted in to the city-wide database for storage following the assessment.

### 2.4 Variables

The variables for this study were selected from four domains of data from the Shanghai Elderly Care Unified Needs Assessment database, as follows:

#### 2.4.1 Basic demographics

Basic demographics included age, gender, education, and cohabitation status. Education was divided into four levels: illiteracy, years of education ≤6 years, 7–12 years, and >12 years. Cohabitation status was divided into two categories: living alone and not living alone.

#### 2.4.2 Living environment

The living environment encompassed the availability of an elevator (or lift) in residence, the indoor steps in residence, the availability of indoor handrails, and the cleanliness and tidiness of residential environment. The cleanliness and tidiness of residential environment were categorized into three levels: clean and tidy, acceptable, and unclean and untidy. These were determined and recorded by the assessors.

#### 2.4.3 Behavioral patterns

Behavioral patterns included housework done by whom, frequency of going outside, and social interactions. Housework fell into three groups as followed: done independently, partially done by others, and all done by others. The frequency of going outside was classified into three levels: daily, sometimes, and hardly. Social interactions were also divided into three levels: good, diminished, and completely lost.

#### 2.4.4 Health conditions

Health conditions included ADL, cognitive function, number of chronic diseases, vision, hearing, number of restricted joints, self-rated quality of sleep and the use of diapers. The ADL was measured by the Barthel Index (28), which is one of the most widely used tools measuring functional independence and ADL. The cut-off value for “Normal” group was 20.0 points, “Mild disability” group was 12.1–19.9 points, “Moderate disability” group was 8.1–12.0 points, and “Severe disability” group was 0–8.0 points. The group of severe disability had been excluded during the inclusion process. Cognitive function was evaluated by the Mini-Mental State Examination (MMSE) (29), which is also a widely used assessment instrument to screen the cognitive impairments. Based on educational levels, the cut-off value for MCI was set as 18–20 points (illiteracy), 21–23 points (year of education ≤6 years), and 25–27 points (year of education >7 years). The cut-off value for dementia was defined as ≤17 points (illiteracy), ≤20 points (year of education ≤6 years), and ≤24 points (year of education >7 years). In this context, MCI and dementia were referred to as cognitive impairment. Disease assessment included 11 chronic diseases, with high prevalences among the older adult population in Shanghai, including chronic obstructive pulmonary disease (COPD), diabetes, chronic pneumonia, lower limb fracture, Parkinson’s disease, intracerebral hemorrhage (ICH), hypertension, advanced tumor, cerebral infarction, coronary atherosclerotic heart disease and Alzheimer’s Disease (AD). The chronic diseases were reviewed and recorded based on the older adult’s medical records by the family doctor. Vision and hearing were divided into two categories each. Vision was deemed normal if it varied from not impaired to partially impaired but with no severely affecting daily life, while abnormal if it ranged from severely affecting daily life to complete blindness. Hearing that ranged from clear perception of normal conversation to perceiving nearby sounds was regarded as normal, whereas inability to perceive nearby sounds to complete deafness was regarded as abnormal. Number of restricted joints referred to the number of joints with more than 50% restriction in joint mobility, including cervical joints, shoulder joint, elbow joint, lumbar joints, hip joint and knee joint. Self-rated quality of sleep referred to the evaluation by older adult regarding their own sleep quality, which is divided into three levels: good, acceptable, and poor.

### 2.5 Statistical analysis

SPSS 23.0 was used for data analysis (30). Firstly, a Chi-squared test (*χ*^2^) was used to compare categorical variables, in the fall group and non-fall group. Then a binary and multivariable logistic regression analyses were conducted sequentially on the correlative factors of falls. Next, a multivariable logistic regression was performed on variables that were significant, stratified by normal cognitive function, MCI and dementia. Multivariable logistic regression is a widely used and well-understood statistical method, which allows for the evaluation of the relationship between multiple independent variables and a single dependent variable. There were two outcome variables: falls occurred in the past 90 days (fall group) and falls not occurred in the past 90 days (not-fall group). *p* < 0.05 was considered as statistically significant.

## 3 Results

### 3.1 General information

The incidence of falls in the past 90 days was 17.67% in this study. Of this, the incidence for cognitively normal older adult was 15.45%, for older adult with MCI was 18.13%, and for older adult with dementia was 18.10%, respectively. Among older adult who fell, 1,970 individuals with normal cognitive function as screened by the MMSE, accounted for 8.98%; 4,340 individuals with MCI accounted for 19.79%, and 15,622 individuals with dementia accounted for 71.23%. As Table 1 presented, there were significant differences between the older adult who had fallen and had not in the past 90 days in terms of basic demographics, living environment, behavioral patterns, and health conditions. In the study population, there were more females than males. The mean age of the older adult who had fallen was 80.1 ± 8.3 years old, which was slightly below the mean age of the older adult who had not fallen (80.6 ± 8.8 years old). However, given that the life expectancy of the older adult in Shanghai in 2022 was 83.18 years (31), the average age of the study population was close to the average life expectancy, and higher than the average age of the general community-dwelling older adults. Among the older adult individuals, 12.1% of those experienced falls and 10.9% of those did not fall lived alone. The data indicates that among the older adult fallers, the proportion lived in residence with elevator or had indoor handrails was lower, while the proportion with indoor steps was higher. In both groups, the vast majority displayed a noticeable decline in ADL, cognitive functions, and poor self-care ability.

**Table 1:** Demographic characteristics of participants.

Characteristics	Fall group (*N* = 21,932)	Not-fall group (*N* = 102,192)	*P*
	*N* (%)	*N* (%)	
Basic demographics			
Gender			0.03
Female	13,541(61.7)	61,947(60.6)	
Male	8,391(38.3)	40,245(39.4)	
Age			<0.001
61–69	2,677(12.2)	13,078(12.8)	
71–79	7,142(32.6)	31,563(30.9)	
≥80	12,113(55.2)	57,551(56.3)	
Education			<0.001
Illiteracy	6,058(27.6)	32,410(31.7)	
≤6 years	4,711(21.5)	19,897(19.5)	
7–12 years	7,827(35.7)	36,224(35.4)	
﹥12 years	1,708(7.8)	7,748(7.6)	
Cohabitation status			<0.001
Living alone (Yes)	2,653(12.1)	11,168(10.9)	
Living environment			
With lift (Yes)	6,850(31.2)	33,815(33.1)	0.01
With indoor steps (Yes)	2,159(9.8)	7,620(7.5)	<0.001
With indoor handrail (Yes)	1,845(8.4)	8,856(8.7)	<0.001
Cleanliness and tidiness of living environment			<0.001
Clean and tidy	468(2.1)	1,306(1.3)	
Acceptable	12,587(57.4)	58,409(57.1)	
Unclean and untidy	8,877(40.5)	42,477(41.6)	
Behavioral patterns			
Housework			<0.001
Done independently	218(1.0)	1,655(1.6)	
Partially done by others	302(1.4)	1,563(1.5)	
All done by others	19,754(90.1)	92,734(90.7)	
Going outside			<0.001
Daily	332(1.5)	2,714(2.7)	
Sometimes	6,254(28.5)	34,636(33.9)	
Hardly	15,346(70.0)	64,842(63.4)	
Social interactions			<0.001
Good	13,259(60.5)	70,028(68.6)	
Diminished	8,254(37.6)	29,746(29.1)	
Completely lose	415(1.9)	2,400(2.3)	
Health conditions			
ADL			<0.001
Normal	123(0.6)	1,318(1.3)	
Mild disability	13,750(62.7)	69,395(67.9)	
Moderate disability	8,059(36.7)	31,479(30.8)	
Cognitive functions			<0.001
Normal	3,189(14.5)	17,453(17.1)	
MCI	7,150(32.6)	32,279(31.6)	
Dementia	11,593(52.9)	52,460(51.3)	
Chronic diseases			<0.001
0	3,490(15.9)	21,658(21.2)	
1	7,637(34.9)	36,389(35.6)	
2	5,667(25.8)	25,589(25.0)	
≥3	5,138(23.4)	18,556(18.2)	
Restricted joints			<0.001
0	13,862(63.2)	70,433(68.9)	
1	5,114(23.3)	20,740(20.3)	
2	1,942(8.9)	7,167(7.0)	
≥3	1,014(4.6)	3,852(3.8)	
Vision (normal)	18,837(85.9)	89,650(87.7)	<0.001
Hearing (normal)	17,653(80.5)	83,438(81.6)	<0.001
Quality of sleep			<0.001
Good	481(2.2)	1,865(1.8)	
Acceptable	7,281(33.2)	37,860(37.1)	
Poor	14,170(64.6)	62,467(61.1)	
Diaper use (Yes)	4,609(21.0)	18,509(18.1)	<0.001

### 3.2 Correlative factors of falls

Initially, a binary logistic regression was employed to analyze a range of factors that may be correlated with falls within the past 90 days, followed by a multivariable logistic regression analysis for those factors that demonstrated significant impact. Table 2 exhibited that, according to the basic demographics, being male and advanced age were found to have a negative correlation with fall occurrences, whereas achieving a higher education level and living independently were positively associated with the likelihood of falls. Specifically, older adult with more than 12 years of education faced a 1.2-fold higher fall risk compared to illiteracy (OR = 1.20, 95% CI = 1.14–1.25, *p* < 0.001).

**Table 2:** Multivariable logistic regression analysis of falls factors.

Characteristics	Fall group		
	OR	95%*CI*	*P*
*Basic demographics*			
Gender			
Female	1	–	–
Male	0.90	0.87–0.93	**<0.001**
Age			
61–69	1	–	–
71–79	0.99	0.94–1.04	0.70
≥80	0.92	0.87–0.96	**0.001**
Education			
Illiteracy	1	–	–
≤6 years	1.05	1.00–1.10	**0.03**
7–12 years	1.15	1.07–1.22	**<0.001**
>12 years	1.20	1.14–1.25	**<0.001**
Cohabitation status			
Living with others	1	–	–
Living alone	1.05	1.00–1.10	**0.04**
Living environment			
With lift (Yes)	0.92	0.89–0.95	**<0.001**
With indoor steps (Yes)	1.38	1.31–1.46	**<0.001**
With indoor handrail (Yes)	0.98	0.92–1.05	0.54
Cleanliness and tidiness of living environment			
Clean and tidy	1	–	–
Acceptable	1.03	1.00–1.07	0.06
Unclean and untidy	1.51	1.35–1.69	**<0.001**
*Behavioral patterns*			
Housework			
Done independently	1	–	–
Partially done by others	0.60	0.52–0.70	**<0.001**
All done by others	0.63	0.56–0.69	**<0.001**
Going outside			
Daily	1	–	–
Sometimes	0.90	0.82–1.00	0.05
Hardly	1.24	1.12–1.37	**<0.001**
Social interactions			
Good	1	–	–
Diminished	1.33	1.29–1.38	**<0.001**
Completely lose	1.07	0.90–1.28	0.46
*Health conditions*			
ADL			
Normal	1	–	–
Mild disability	0.39	0.34–0.44	**<0.001**
Moderate disability	0.37	0.33–0.42	**<0.001**
Cognitive functions			
Normal	1	–	–
MCI	1.08	1.03–1.13	**0.002**
Dementia	1.06	0.01–1.11	**0.03**
Chronic diseases			
0	1	–	**–**
1	1.50	1.42–1.57	**<0.001**
2	1.47	1.40–1.56	**<0.001**
≥3	1.76	1.66–1.86	**<0.001**
Restricted joints			
0	1	–	**–**
1	1.23	1.18–1.28	**<0.001**
2	1.35	1.28–1.44	**<0.001**
≥3	1.49	1.37–1.63	**<0.001**
Vision (impaired)	1.06	1.01–1.11	**0.02**
Hearing (impaired)	0.97	0.93–1.02	0.22
Quality of sleep			
Good	1	–	–
Acceptable	0.37	0.34–0.40	**<0.001**
Poor	0.41	0.38–0.45	**<0.001**
Diaper use (Yes)	1.28	1.23–1.35	**<0.001**

The bold values provided in Tables 2, 3 refers to P value less than 0.05.

Regarding the living environment, residences with lift was inversely correlated with falls (OR = 0.92, 95%CI = 0.89–0.95, *p* < 0.001), whereas residence with indoor steps was positively related to falls (OR = 1.38, 95%CI = 1.31–1.46, *P* < 0.001). Additionally, the likelihood of falling for older adult living in unclean and cluttered environment was 1.51 times (OR = 1.51, 95%CI = 1.35–1.69, *P* < 0.001) that of those living in clean and orderly conditions.

Behavioral patterns suggested that when housework were performed by someone else (whether partially or in whole), there was a correlation with a lower risk of falls. Conversely, limited outdoor activities (hardly going outside) and diminished social interactions were linked to an increased risk of falling. Specifically, the risk of falling for the older adult who hardly went outside was 1.24 times higher (OR = 1.24, 95%CI = 1.12–1.37, *p* < 0.001) than for those who went outside daily. Likewise, older adult with diminished social interactions had a 1.33 times higher fall risk (OR = 1.33, 95%CI = 1.29–1.38, *p* < 0.001) compared to those maintaining good and robust social interactions.

Concerning health conditions, compromised ADL and either acceptable or poor sleep quality showed a negative association with fall incidents. Furthermore, declined cognitive functions, chronic diseases, restricted joints, poor vision, and the use of diapers were positively associated with the likelihood of falls. Older adult with MCI and dementia exhibited a greater tendency to fall compared to those with normal cognitive functions. There was a notable positive correlation between the increase in chronic diseases and mobility limitations in joints on the one hand, and fall risk on the other. Notably, older adult with three or more chronic diseases showed the most significant correlation, facing a greater odds ratio of 1.76 (OR = 1.76, 95%CI = 1.66–1.86, *p* < 0.001), compared to older adults without chronic diseases. Similarly, those with three or more mobility-restricted joints were 1.49 times (OR = 1.49, 95%CI = 1.37–1.63, *p* < 0.001) more likely to fall than their counterparts with no joint restrictions. Vision acted as a marginally protective factor. Older adult with impaired vision or those requiring the use of diapers were at an increased risk of experiencing falls.

### 3.3 Correlative factors of falls in older adult with cognitive impairment

Multivariable logistic regression was conducted on significant variables identified in Table 2, with three individual models for normal cognitive functions, MCI and dementia group, respectively. Table 3 illustrated that, regarding the basic demographics, older adult men had a lower risk of falling compared to older adult women. There was a slightly increased risk of falls among older adult men with MCI and dementia, as opposed to those with normal cognitive functions. The risk of falls in advanced older adult (aged 80 years and above) with MCI was marginally higher than that in advanced older adult with normal cognitive functions. Educational level was positively correlated with fall risk in older adults with dementia. Older adult with dementia who lived alone were facing a fall risk that was 1.191 times higher (OR = 1.191, 95%CI = 1.109–1.278, *p* < 0.001) than their counterparts residing with others. However, no significant association was found between living alone and falls either in the older adult with normal cognitive functions or MCI.

**Table 3:** Multivariable logistic regression analysis of falls factors by cognitive functions.

Characteristics	Fall group		
	Normal	MCI	Dementia
	OR (95%*CI*)	OR (95%*CI*)	OR (95%*CI*)
*Basic demographics*			
Gender			
Female	1	1	1
Male	**0.809(0.745–0.877)**	**0.940(0.889–0.995)**	**0.919(0.878–0.962)**
Age			
61–69	1	1	1
71–79	0.959(0.871–1.057)	0.998(0.921–1.082)	1.049(0.963–1.143)
≥80	**0.803(0.719–0.897)**	**0.872(0.804–0.947)**	1.025(0.945–1.111)
Education			
Illiteracy	1	1	1
≤6 years	**0.543(0.450–0.655)**	1.012(0.930–1.101)	**1.353(1.280–1.430)**
7–12 years	**0.419(0.354–0.497)**	0.925(0.853–1.004)	**1.317(1.239–1.400)**
>12 years	**0.441(0.362–0.537)**	1.005(0.897–1.126)	**1.420(1.287–1.567)**
Cohabitation status (living alone)	1.002(0.903–1.112)	1.037(0.959–1.120)	**1.191(1.109–1.278)**
Living environment			
With lift (Yes)	**0.881(0.811–0.958)**	0.979(0.921–1.040)	**0.901(0.857–0.948)**
With indoor steps (Yes)	1.154(0.968–1.375)	0.944(0.846–1.053)	**1.591(1.492–1.695)**
Cleanliness and tidiness of living environment			
Clean and tidy	1	1	1
Acceptable	1.008(0.929–1.093)	**1.137(1.074–1.202)**	0.999(0.952–1.048)
Unclean and untidy	**1.545(1.151–2.073)**	**1.774(1.424–2.210)**	**1.374(1.180–1.599)**
*Behavioral patterns*			
Housework			
Done independently	1	1	1
Partially done by others	1.008(0.929–1.093)	**0.681(0.536–0.864)**	**0.429(0.321–0.574)**
All done by others	0.854(0.707–1.033)	**0.698(0.581–0.838)**	**0.464(0.377–0.570)**
Going outside			
Daily	1	1	1
Sometimes	**1.254(1.048–1.502)**	**0.795(0.666–0.949)**	**0.742(0.617–0.892)**
Hardly	**1.600(1.334–1.920)**	**1.206(1.010–1.440)**	0.965(0.804–1.158)
*Social interactions*			
Good	1	1	1
Diminished	**1.394(1.262–1.539)**	**1.240(1.164–1.321)**	**1.355(1.293–1.419)**
Completely lose	**2.395(1.249–4.591)**	0.681(0.434–1.071)	1.143(0.931–1.403)
*Health conditions*			
ADL			
Normal	1	1	1
Mild disability	**0.566(0.462–0.694)**	**0.368(0.295–0.459)**	**0.354(0.275–0.455)**
Moderate disability	**0.700(0.559–0.878)**	**0.445(0.353–0.561)**	**0.320(0.248–0.413)**
Chronic diseases			
0	1	1	1
1	**1.645(1.464–1.848)**	**1.478(1.349–1.619)**	**1.581(1.468–1.703)**
2	**1.311(1.158–1.484)**	**1.450(1.319–1.593)**	**1.663(1.537–1.798)**
≥3	**1.873(1.639–2.139)**	**1.773(1.609–1.955)**	**1.817(1.674–1.974)**
Restricted joints			
0	1	1	1
1	**1.783(1.444–2.202)**	1.143(0.983–1.329)	**1.628(1.435–1.848)**
2	**1.597(1.398–1.825)**	**1.226(1.111–1.352)**	**1.308(1.194–1.432)**
≥3	**1.436(1.308–1.577)**	0.995(0.929–1.065)	**1.324(1.252–1.400)**
Vision (impaired)	1.014(0.891–1.153)	**1.096(1.008–1.192)**	1.008(0.950–1.071)
Quality of sleep			
Good	1	1	1
Acceptable	**0.323(0.272–0.384)**	**0.461(0.394–0.540)**	**0.569(0.494–0.654)**
Poor	**0.341(0.286–0.407)**	**0.479(0.409–0.561)**	**0.641(0.557–0.737)**
Diaper use (Yes)	**1.297(1.119–1.503)**	**1.270(1.163–1.387)**	**1.302(1.225–1.383)**

Each group was analyzed individually using a multivariable logistic regression.

The bold values provided in Tables 2, 3 refers to P value less than 0.05.

From an environmental perspective, compared to those living in residences without lift, both older adults with normal cognitive functions and dementia living in residences with lift exhibited a reduced fall risk, with older adult with normal cognitive functions experiencing a marginally lower risk than those suffering from dementia. Older adult with dementia residing in environments with steps faced a remarkable higher risk of falling (OR = 1.591, 95%CI = 1.492–1.695, *P* < 0.001) compared to those in step-free settings among three groups. However, this factor did not show significant correlation with fall occurrences among older adult with normal cognitive functions and MCI. Relative to living in clean and tidy surroundings, the fall risk was higher in all three groups who living in unclean and cluttered environments, with those suffered from MCI encountering the highest increased risk (OR = 1.774, 95%CI = 1.424–2.210, *P* < 0.001).

In regard to behavioral patterns, older adult with MCI and dementia, who had the assistance of housework from others (some or all), showed a reduced risk of falling compared to those performing housework independently. This correlation between housework and fall risk was not significant among older adult with normal cognitive functions. Compared to daily outings, for older adult with normal cognitive functions, a reduced frequency of going outside was positively correlated with an increased risk of falling. Yet, for older adult with MCI, sometimes going outside was negatively correlated with the risk of falling, whereas hardly going outside was positively correlated with an increased risk of falling. For older adult with dementia, sometimes going outside was also negatively correlated with the risk of falling. Furthermore, comparing with older adult with good social interactions, those with diminished social interactions experienced heightened fall risks, which was particularly notable within the cognitively normal group. The risk of falls for older adult with normal cognitive functions who completely lost social interactions was 2.395 times (OR = 2.395, 95%CI = 1.249–4.591, *p* = 0.009).

From the perspective of health conditions, both ADL and quality of sleep were found inversely associated with fall risk, whereas the presence of chronic diseases, joint restrictions, impaired vision, and diapers use were positively associated with fall incidents. Compared with older adult with normal ADL, older adult with mild or moderate disability across all three groups demonstrated a lower risk of falls. Nevertheless, those suffering from chronic diseases and joint restrictions faced a higher risk of falling. Moreover, among the three groups, those using diapers faced a higher risk of falling, with older adult with dementia who used diapers showing the highest fall risk (OR = 1.302, 95%CI = 1.225–1.383, *P* < 0.001).

## 4 Discussion

This study was based on a cross-sectional survey with a large sample size of 179,141 individuals, 124,124 participants were finally included, among whom the fall incidence in the past 90 days was 17.67%. This result is higher than the incidences of falls reported in some previous studies in China (32, 33). Despite numerous studies investigating the risk factors for falls among the older adult, the exploration of these factors specifically for individuals with dementia, particularly in large-scale studies, remains limited. Research has shown that older adult at risk of falls encompass those with MCI and dementia. Targeted fall prevention strategies designed for this distinct group may contribute to a decrease in the incidence of falls (21). The study initially conducted both binary and multivariable logistic regression analyses to explore the relationship between fall risk and four categories of factors: basic demographics, living environment, behavioral patterns and health conditions. Subsequently, multivariable logistic regression was carried out on variables that were significant, stratified by cognitive function levels.

It was found that women had an increased risk of experiencing falls compared to men, which aligned with the conclusions drawn in previous studies (18, 32, 34). In addition, significant factors inversely associated with falls include: advanced age, mild to moderate of disability, and ranging from acceptable to poor sleep quality. Conversely, main correlative factors positively related to falls encompassed: higher education level, the presence of indoor steps in residence, an unclean and untidy living environment, hardly going outside, diminished social interactions, chronic diseases, restricted joints, and the use of diaper. Advanced older adult with mild to moderate disability were more likely to exhibit poor self-care and physical capabilities, thus being incapable of performing housework independently.

Further, advanced age, residence with indoor steps, unclean and untidy living environment, impaired vision and the use of diaper were positively related to fall in older adult with cognitive impairments. It was reported that the lack of handrails was considered as an extrinsic risk factor (26), while the result was not significant in this study. The literature supported that dementia contribute to the significantly elevated risk of falls (35). Some researches also outlined a significant correlation between reduced cognitive functions, slower walking speed, and delayed reaction times, which consequently led to an increased incidence of falls (36–38).

In terms of basic demographics, among the cognitively normal older adult, education level was negatively related to fall, and which was inconsistent with previous findings, (39) whereas among the older adult with dementia, the correlation was opposite. Taking older adult with >12 years of education as an example, among older adult with dementia and an educational level of >12 years, the proportion of those aged 80 and above accounted 72.1%, while in the cognitively normal older adult with >12 years of education, the proportion of those aged 80 and above was 43.5%, which was significantly lower than that in older adult with dementia. The potential reason for this observed result could be that within the older adult with dementia, individuals with a higher level of education tend to be older and, therefore, have relatively poorer self-care abilities. Additionally, living alone was found as a correlative factor for falls, and older adult with dementia who lived alone was positively related to fall. It was advisable for older adults with cognitive impairments to cohabit with others.

In terms of living environment, the presence of lift in residence was negatively related to falls among older adult with normal cognitive functions and dementia, whereas having indoor steps was a significant positively correlative factor for falls among older adult with dementia. It is evident that maintaining an accessible living environment can effectively reduce the risk of falls in older adult with dementia. Moreover, in all three categorized groups, an unclean and untidy environment was reported as a positive correlated factor with the risk of falling, yet the highest at risk group was the older adult with MCI group living in unclean and untidy environment, followed by normal older adult, and older adult with dementia. This is a counter-intuitive result, which could be due to other people assisting or protecting the older adult with dementia from falls. Therefore, creating and maintaining the cleanness and tidiness of living space was negatively related to fall for older adults with MCI. Studies revealed that the living environment was a protective factor in preventing falls, prompting us to arrange a suitable living environment for the older adult to reduce their safety hazards, and prevent them from feeling stressed by environmental stimulus (32).

In the aspect of behavioral patterns, for older adult with normal cognitive functions, going outside daily and maintaining good social interactions were both negatively related to fall. The correlation between social interaction and falls was more pronounced in the general older adult population. However, for older adult with MCI and dementia, going outside sometimes and maintaining good social interaction was found negatively correlated with the risk of falling. This results indicated that outdoor activities of normal older adult and older adult with cognitive impairment may need to be treated differently, and more longitudinal cohort studies are needed to determine causal effects. Evidence from previous researches supports that lower levels of weekly walking activity were significantly associated with an increased risk of falls (23). Especially in comparison to older adult with MCI and dementia, encouraging daily outings and maintaining good social functioning is crucial for preventing falls for general older adults. The relationship between the level of physical activity and fall risk was U-shaped, suggested in a previous study, meaning the preventive effects of activity or exercise may be offset by an increased likelihood of falls among highly active population (40). Given that older adult with cognitive impairments may have diminished control over physical movements (40), it is particularly advisable for older adult with cognitive impairments to go outside in an appropriate frequency.

Older adults with cognitive impairment had higher risk of falls in those with MCI, which is consistent with previous literature (41). The hypothesis suggesting that disability serves as a factor inversely related to fall risks can attributed to the observation that older adult tend to participate less in physical activities. This lack of engagement in physical activities subsequently led to a comparatively lower risk of falls. The ADL and quality of sleep were found to be inversely correlated with fall risk among three groups of older adult. According to previous researches, frailty was related to aging, which often led to an increased risk of falling (42), meanwhile frailty disturbed the sleep cycle and linked to sleep disorders (43). The negative correlation between either ADL or quality of sleep, and falls, may have a hypothesized link between poor sleep quality and falls. Additional studies are needed to explore this association further. Moreover, the presence of multiple chronic diseases and restricted joints were both found to have a significant positive correlation with the likelihood of falls among all three groups. Previous research has likewise verified that chronic health issues can increase the likelihood of falls, including hypertension, diabetes, stroke, Parkinson’s disease, etc., some of which were investigated in this study (39, 44, 45). It was also frequently found in the literature that falls in community-dwelling older adults was significantly associated with instability (46–48). As joint limitations were found to exacerbate instability, this positive association indicated that managing the coexisting conditions and addressing joint limitations effectively could substantially reduce the risk of falls among the older adult. For older adults, particularly those with cognitive impairments who rely on the use of diapers, caregivers need to pay more attention to whether there is any inconvenience in movement.

The strength of this study lies in observation of characteristics of falls among the older adult with cognitive impairments. Moreover, it was based on big data, and the sample size was very large and thus with good representative. However, this study has some limitations. Firstly, the research was a cross-sectional study, which only indicated associations between factors and risk of falls, cannot elucidate the causal relationships. Secondly, the participants were individuals who voluntarily apply for Long-term Care Insurance in Shanghai, and hence they all had certain care needs to some extent. Compared to the general community-dwelling older adult, participants in this study tended to be older and were in relatively poorer health. Thirdly, to eliminate the influence of psychotropic medications on falls, the study excluded older adult diagnosed with dementia by professional psychiatrists, and used MMSE to classify the levels of cognitive functions. The MMSE served as a screening tool, not a diagnostic standard for MCI and dementia.

## 5 Conclusion

This study was a cross-sectional survey with a large study population of 124,124 older adult individuals included. This study explored the correlative factors of falls among the older adult with cognitive impairment, to provide distinct evidence for preventing falls in the older adult with cognitive impairment compared with the general older adult population. Optimal outdoor activity plans should be developed separately based on the cognitive function of older adults. In addition, attention should be paid to co-existing chronic conditions and environmental hazards. The findings within this study prompt to consider the need for distinct interventions in fall prevention for the older adult with MCI and dementia. Further research is necessary to delve deeper, and to validate the findings across a broader population.

## Data availability statement

The original contributions presented in the study are included in the article/supplementary material, further inquiries can be directed to the corresponding authors.

## Ethics statement

Written informed consent was obtained from the individual(s) for the publication of any potentially identifiable images or data included in this article.

## Author contributions

CW: Conceptualization, Methodology, Writing – original draft, Writing – review & editing, Investigation. YZ: Data curation, Investigation, Writing – original draft, Methodology, Software, Writing – review & editing. JW: Investigation, Writing – review & editing. LW: Formal analysis, Writing – review & editing. BL: Data curation, Writing – original draft, Formal analysis, Writing – review & editing. HD: Conceptualization, Data curation, Funding acquisition, Investigation, Methodology, Project administration, Resources, Supervision, Validation, Writing – review & editing.

## References

[1] WHO. Falls. (2021). Available at: https://www.who.int/news-room/fact-sheets/detail/falls (Accessed April 26, 2021).

[2] Deandrea S Lucenteforte E Bravi F Foschi R La Veccha C Negri E. Risk factors for falls in community- dwelling older people: a systematic review and meta-analysis. Epidemiology. (2010) 21:658–68. doi: 10.1097/ede.0b013e3181e89905 20585256

[3] Miyoshi Y Kondo Y Hirano Y Ishihara T Sueyoshi K Okamoto K. Characteristics, injuries, and clinical outcomes of geriatric trauma patients in Japan: an analysis of the nationwide trauma registry database. Sci Rep. (2020) 10:19148–6. doi: 10.1038/s41598-020-76149-4, PMID: 33154440 PMC7645585

[4] Ang GC Low SL How CH. Approach to falls among the elderly in the community. Singapore Med J. (2020) 61:116–21. doi: 10.11622/smedj.2020029, PMID: 32488276 PMC7905119

[5] Kim M Chang M Nam E Kim SG Cho SI Ryu DH. Fall characteristics among elderly populations in urban and rural areas in Korea. Medicine. (2020) 99:e23106. doi: 10.1097/MD.0000000000023106 33181676 PMC7668504

[6] Florence CS Bergen G Atherly A Burns E Stevens J Drake C. Medical costs of fatal and non-fatal falls in older adults: medical costs of falls. J Am Geriatr Soc. (2018) 66:693–8. doi: 10.1111/jgs.15304, PMID: 29512120 PMC6089380

[7] Vongsachang H Mihailovic A E JY Friedman DS West SK Gitlin LN. The impact of weather and seasons on falls and physical activity among older adults with Glaucoma: a longitudinal prospective cohort study. Sensors. (2021) 21:3415. doi: 10.3390/s21103415, PMID: 34068938 PMC8156454

[8] China Association of Geriatric Health Care Medicine Subcommittee on Geriatric Health Services and Standardization, Editorial Committee of Chinese Journal of Geriatric Care. Chinese consensus on fall risk assessment for the elderly (draft proposal). Chin J Geriatr Care. (2019) 17:47–48, 50. doi: 10.3969/j.issn.1672-2671.2019.04.013

[9] E J-Y Mihailovic A Schrack JA Li T Friedman DS West SK. Characterizing longitudinal changes in physical activity and fear of falling after falls in Glaucoma. J Am Geriatr Soc. (2021) 69:1249–56. doi: 10.1111/jgs.17014, PMID: 33418602 PMC8302884

[10] World Health Organization. Dementia. (2023). Available at: https://www.who.int/news-room/fact-sheets/detail/dementia (Accessed March 15, 2023).

[11] Gale SA Acar D Daffner KR. Dementia. Am J Med. (2018) 131:1161–9. doi: 10.1016/j.amjmed.2018.01.022 29425707

[12] Petersen RC. Clinical practice. Mild cognitive impairment. N Engl J Med. (2011) 364:2227–34. doi: 10.1056/NEJMcp0910237 21651394

[13] Winblad B Palmer K Kivipelto M Jelic V Fratiglioni L Wahlund LO. Mild cognitive impairment--beyond controversies, towards a consensus: report of the international working group on mild cognitive impairment. J Intern Med. (2004) 256:240–6. doi: 10.1111/j.1365-2796.2004.01380.x 15324367

[14] Douglas A Letts L Richardson J. A systematic review of accidental injury from fire, wandering and medication self-administration errors for older adults with and without dementia. Arch Gerontol Geriatr. (2011) 52:e1–e10. doi: 10.1016/j.archger.2010.02.014, PMID: 20334937

[15] Wang J Xiao LD Wang K Luo Y Li X. Cognitive impairment and associated factors in rural elderly in North China. J Alzheimers Dis. (2020) 77:1241–53. doi: 10.3233/JAD-200404, PMID: 32925043

[16] Härlein J Halfens RJG Dassen T Lahmann NA. Falls in older hospital inpatients and the effect of cognitive impairment: a secondary analysis of prevalence studies. J Clin Nurs. (2011) 20:175–83. doi: 10.1111/j.1365-2702.2010.03460.x, PMID: 21158990

[17] Muir SW Gopaul K Montero Odasso MM. The role of cognitive impairment in fall risk among older adults: a systematic review and meta-analysis. Age Ageing. (2012) 41:299–308. doi: 10.1093/ageing/afs012, PMID: 22374645

[18] Meuleners LB Fraser ML Bulsara MK Chow K Ng JQ. Risk factors for recurrent injurious falls that require hospitalization for older adults with dementia: a population based study. BMC Neurol. (2016) 16:188. doi: 10.1186/s12883-016-0711-3, PMID: 27687085 PMC5041288

[19] Lim SC Mamun K Lim JK. Comparison between elderly inpatient fallers with and without dementia. Singapore Med J. (2014) 55:67–71. doi: 10.11622/smedj.2014017, PMID: 24570314 PMC4291931

[20] Kröpelin TF Neyens JC Halfens RJ Kempen GI Hamers JP. Fall determinants in older long-term care residents with dementia: a systematic review. Int Psychogeriatr. (2013) 25:549–63. doi: 10.1017/S1041610212001937, PMID: 23253253

[21] Lach HW Harrison BE Phongphanngam S. Falls and fall prevention in older adults with early-stage dementia: an integrative review. Res Gerontol Nurs. (2017) 10:139–48. doi: 10.3928/19404921-20160908-01 27665756

[22] Ren Z Li Y Li X Shi H Zhao H He M. Associations of body mass index, waist circumference and waist-to-height ratio with cognitive impairment among Chinese older adults: based on the CLHLS. J Affect Disord. (2021) 295:463–70. doi: 10.1016/j.jad.2021.08.093, PMID: 34507227

[23] Chantanachai T Taylor ME Lord SR Menant J Delbaere K Sachdev PS. Risk factors for falls in community-dwelling older people with mild cognitive impairment: a prospective one-year study. Peer J. (2022) 10:e13484. doi: 10.7717/peerj.13484 35663527 PMC9161814

[24] Santiago Martinez P Lord SR Close JCT Taylor ME. Associations between psychotropic and anti-dementia medication use and falls in community-dwelling older adults with cognitive impairment. Arch Gerontol Geriatr. (2023) 114:105105. doi: 10.1016/j.archger.2023.105105, PMID: 37364485

[25] Shao L Shi Y Xie XY Wang Z Wang ZA Zhang JE. Incidence and risk factors of falls among older people in nursing homes: systematic review and Meta-analysis. J Am Med Dir Assoc. (2023) 24:1708–17. doi: 10.1016/j.jamda.2023.06.002 37433427

[26] de Smet L Carpels A Creten L De Pauw L Van Eldere L Desplenter F. Prevalence and characteristics of registered falls in a Belgian university psychiatric hospital. Front Public Health. (2022) 10:1020975. doi: 10.3389/fpubh.2022.1020975, PMID: 36388388 PMC9651969

[27] Norman KJ Hirdes JP. Evaluation of the predictive accuracy of the interRAI falls clinical assessment protocol, Scott fall risk screen, and a supplementary falls risk assessment tool used in residential long-term care: a retrospective cohort study. Can J Aging. (2020) 39:521–32. doi: 10.1017/S0714980820000021, PMID: 32172692

[28] Lawton MP Brody EM. Assessment of older people: self-maintaining and instrumental activities of daily living. Gerontologist. (1969) 9:179–86. doi: 10.1093/geront/9.3_Part_1.179, PMID: 5349366

[29] Folstein MF Folstein SE McHugh PR. “Mini-mental state”. A practical method for grading the cognitive state of patients for the clinician. J Psychiatr Res. (1975) 12:189–98. doi: 10.1016/0022-3956(75)90026-6 1202204

[30] IBM Corp. IBM SPSS statistics for windows, version 23.0. Armonk, NY: IBM Corp (2015).

[31] Shanghai Bureau of Statistics. Shanghai Statistical Yearbook (2023). Available at: https://tjj.sh.gov.cn/tjnj/nj23.htm?d1=2023tjnjen/E0201.htm (accessed April 28, 2024.

[32] Dong X Liu G Yin X Min R Hu Y. Fall risks and the related factors for the homebound older people with dementia: evidence from East China. Front Public Health. (2022) 10:946097. doi: 10.3389/fpubh.2022.946097, PMID: 36091547 PMC9458357

[33] ZhangYJFuSHZhuQNingCXLuanFXZhangF. Underweight in men had a closer relationship with falls than women in centenarians. J Nutr Health Aging. (2020) 24:987–92. doi: 10.1007/s12603-020-1508-z, PMID: 33155626

[34] Bally ELS Ye L van Grieken A Tan SS Mattace-Raso F Procaccini E. Factors associated with falls among hospitalized and community-dwelling older adults: the APPCARE study. Front Public Health. (2023) 11:1180914. doi: 10.3389/fpubh.2023.1180914, PMID: 37457268 PMC10344358

[35] Taylor ME Close JCT. Dementia. Handb Clin Neurol. (2018) 159:303–21. doi: 10.1016/B978-0-444-63916-5.00019-7 30482323

[36] Taylor ME Delbaere K Lord SR Mikolaizak AS Close JC. Physical impairments in cognitively impaired older people: implications for risk of falls. Int Psychogeriatr. (2013) 25:148–56. doi: 10.1017/S1041610212001184 22831907

[37] Dyer AH Lawlor B Kennelly SP NILVAD Study Group. Gait speed, cognition and falls in people living with mild-to-moderate Alzheimer disease: data from NILVAD. BMC Geriatr. (2020) 20:117. doi: 10.1186/s12877-020-01531-w, PMID: 32228468 PMC7106668

[38] Taylor ME Delbaere K Lord SR Mikolaizak AS Brodaty H Close JC. Neuropsychological, physical, and functional mobility measures associated with falls in cognitively impaired older adults. J Gerontol A Biol Sci Med Sci. (2014) 69:987–95. doi: 10.1093/gerona/glt166, PMID: 24149433

[39] Xu Q Ou X Li J. The risk of falls among the aging population: a systematic review and meta-analysis. Front Public Health. (2022) 10:902599. doi: 10.3389/fpubh.2022.902599, PMID: 36324472 PMC9618649

[40] Kamimura S Iida T Watanabe Y Kitamura K Kabasawa K Takahashi A. Physical activity and recurrent fall risk in community-dwelling Japanese people aged 40-74 years: the Murakami cohort study. Eur Rev Aging Phys Act. (2022) 19:20. doi: 10.1186/s11556-022-00300-5 36056330 PMC9438326

[41] Okoye SM Fabius CD Reider L Wolff JL. Predictors of falls in older adults with and without dementia. Alzheimers Dement. (2023) 19:2888–97. doi: 10.1002/alz.12916, PMID: 36633222 PMC10336176

[42] Alqahtani BA. Association between physical frailty and sleep quality among Saudi older adults: a community-based, cross-sectional study. Int J Environ Res Public Health. (2021) 18:12741. doi: 10.3390/ijerph182312741, PMID: 34886467 PMC8656802

[43] Wai JL Yu DS. The relationship between sleep-wake disturbances and frailty among older adults: a systematic review. J Adv Nurs. (2020) 76:96–108. doi: 10.1111/jan.14231, PMID: 31588595

[44] Barik M Panda SN Tripathy SS Sinha A Ghosal S Acharya AS. Is multimorbidity associated with higher risk of falls among older adults in India? BMC Geriatr. (2022) 22:486. doi: 10.1186/s12877-022-03158-5, PMID: 35658840 PMC9167508

[45] Paliwal Y Slattum PW Ratliff SM. Chronic health conditions as a risk factor for falls among the community-dwelling US older adults: a zero-inflated regression modeling approach. Biomed Res Int. (2017) 2017:5146378–9. doi: 10.1155/2017/5146378, PMID: 28459060 PMC5387801

[46] Sousa LM Marques-Vieira CM Caldevilla MN Henriques CM Severino SS Caldeira SM. Risk for falls among community-dwelling older people: systematic literature review. Rev Gaucha Enferm. (2017) 37:e55030. doi: 10.1590/1983-1447.2016.04.55030 28273251

[47] Chantanachai T Sturnieks DL Lord SR Payne N Webster L Taylor ME. Risk factors for falls in older people with cognitive impairment living in the community: systematic review and meta-analysis. Ageing Res Rev. (2021) 71:101452. doi: 10.1016/j.arr.2021.101452 34450352

[48] Abdul Rahman K Ahmad SA Che Soh A Ashari A Wada C Gopalai AA. The Association of Falls with instability: an analysis of perceptions and expectations toward the use of fall detection devices among older adults in Malaysia. Front Public Health. (2021) 9:612538. doi: 10.3389/fpubh.2021.612538, PMID: 33681130 PMC7928312

